# Urinary KIM-1 is not correlated with gestational age among 5-year-old children born prematurely

**DOI:** 10.3389/fped.2023.1038206

**Published:** 2023-03-20

**Authors:** Jaime M. Restrepo, Laura Torres-Canchala, Joseph V. Bonventre, Juan C. Arias, Michael Ferguson, Adriana Villegas, Oscar Ramirez, Guido Filler

**Affiliations:** ^1^ Pediatric Nephrology Service, Fundación Valle del Lili, Cali, Colombia; ^2^ Centro de Investigaciones Clínicas, Fundación Valle del Lili, Cali, Colombia; ^3^ Facultad de Ciencias de la Salud, Universidad Icesi, Cali, Colombia; ^4^ Renal Division, Brigham and Women's Hospital, Boston, MA, United States; ^5^ Kangaroo Mother House Alfa, Cali, Colombia; ^6^ Pediatric Nephrology Service, Boston Children Hospital, Boston, United States; ^7^ Department of Pathology and Laboratory Medicine, Fundación Valle del Lili, Cali, Colombia; ^8^ Department of Pediatrics, Centro Médico Imbanaco de Cali, Cali, Colombia; ^9^ Fundación POHEMA, Cali, Colombia; ^10^ Departments of Paediatrics, Medicine, and Pathology and Laboratory Medicine, University of Western Ontario, London, ON, Canada; ^11^ The Lilibeth Caberto Kidney Clinical Research Unit, Western University, London, ON, Canada

**Keywords:** KIM-1 (kidney injury molecule 1), gestational age (GA), renal volume z-scores, specific gravity (density), prematurity and low birth weight

## Abstract

**Background:**

Preterm birth is associated with decreased nephron endowment. Currently, there is no reliable non-invasive biomarker to identify or monitor decreased nephron number in at-risk patients. Urinary Kidney Injury Molecule-1 (KIM-1) is a biomarker of acute and chronic renal injury. We measured urinary KIM-1 among a wide array of other potential biomarkers.

**Methods:**

We conducted an ambispective cohort study of 5-years-old children born prematurely and healthy controls identified from city schools. Detailed anthropometrics, renal ultrasound dimensions, and biochemical parameters were measured. Urinary KIM-1 was measured using Luminex® technology. Age independent z-scores were calculated and compared. Spearman correlations were used for estimating the association between measures and KIM-1.

**Results:**

We enrolled 129 children, 97 (75.2%) born pre-term and 32 (24.8%) healthy controls born at full-term. Pre-term patients had significantly lower weight and body surface area than controls. Pre-term patients and controls did not differ in current age, sex, race, height, blood pressure, urinary sodium, fractional sodium excretion, serum creatinine and estimated GFR. All spearman correlation between KIM-1 and gestational age, renal and serum measurements were weak without statistical significance

**Conclusion:**

In 5-year-old children born prematurely, KIM-1 was not correlated with gestational age. Further prospective studies need to confirm this finding.

## Introduction

Prematurity leads to increased cardiovascular morbidity and mortality in adulthood (1), necessitating an approach for early diagnosis and intervention (2). It is generally believed that nephrogenesis, which normally continues until 36 weeks of gestation, is prematurely stopped, or at least altered with premature delivery (3, 4). Reduced nephron endowment is considered a risk factor for prenatally programmed adult morbidity, but we currently do not have a good tool for determining nephron endowment *in vivo* (5, 6). Traditionally, renal function is measured using serum creatinine, but creatinine is very insensitive to mildly decreased nephron endowment (3). Renal volumes have been proposed to assess nephron endowment in the neonatal period (7). However, after preterm birth, renal volume of the cortex increases rapidly (8) due to glomerular hyperfiltration (8). Renal volumes of prematurely born Swedish children (<28 weeks of gestation) are not different from healthy controls when corrected to body surface area (9) To identify children at risk, tubular function would have to be assessed, rather than estimate or measure glomerular filtration rate (GFR). In that context, it is postulated that the remaining tubules would have to excrete more urinary sodium and potassium to achieve salt and fluid homeostasis. In adults, the contralateral kidney significantly increased the fractional sodium excretion 90 days after nephrectomy (10). Moreover, urinary calcium excretion should be increased if urinary sodium excretion is increased since urinary calcium reabsorption in the distal tubule is impaired in the presence of a higher urinary sodium concentration (11, 12).

In a recent paper about reduced nephron endowment in a murine model of kidney fibrosis, urinary Kidney-Injury-Molecule-1 (KIM-1) was found to be elevated (13). Ideally, a biomarker of reduced nephron endowment should also be correlated with gestational age. Our study aim was to determine if KIM-1 (14, 15), a marker of tubular injury, would be different between 5-year old children born pre-term, compared to controls who were born at full-term. We hypothesized that urinary sodium excretion and urinary KIM-1 would be higher with a lower gestational age at birth as there are fewer tubules to handle the filtered load.

## Materials and methods

### Setting and study population

This study was performed in Cali, Colombia, using a sample of convenience served at two institutions: “Casa Madre Canguro Alfa” and Fundación Valle de Lili Hospital. “Casa Madre Canguro Alfa” is a nurse-led comprehensive program that provides longitudinal health care for preterm children. Fundación Valle del Lili is a tertiary care university with a pediatric nephology practice that serves 3,000 patients per year and a catchment area of 4.6 million people in the Southwest area of Colombia.

### Study design, inclusion criteria and data collection

This was an ambispective study. The exposed cohort comprised of children who graduated from the “Casa Madre Canguro Alfa” program. Data was collected at 5 years of age (±3 months). The inclusion criteria included prematurity < 37 weeks of gestation and a birth weight < 2,500 grams. We excluded children with major congenital or renal abnormalities and/or those who were lost to follow-up. The enrolled children had their first visit to the Casa Madre Canguro Alfa program between 2006 and 2012. Healthy children with birth weight ≥ 2,500 grams and gestational age ≥ 37 weeks from different schools around the city were enrolled as controls. This study was in accordance with the Declaration of Helsinki and was approved by the Fundación Valle del Lili ethics board committee. Informed written consent was obtained from parents in each case.

### Outcome variables

The main outcome variables were urinary electrolyte concentrations and urinary KIM-1 levels and their respective urinary creatinine ratios at 5 years of age. Furthermore, each child had one renal ultrasound study interpreted by three independent radiologists for renal dimensions (with a General Electric, LOGIQ* E9) in triplicates. An age-appropriate 9–12 mHz curved array transducer was used with the participant lying in the supine position and scanned in the para-coronal view with the transducer positioned to obtain the longest kidney dimension. Kidney volume was calculated using the ellipsoid formula from the average of the 9 measurements in each dimension. Simultaneously, 3 repeated measurements for patient anthropometrics were obtained. One measure of standard serum and urine biochemistry tests were obtained.

Height was measured without shoes on a wall mounted stadiometer and weight was measured without heavy clothes using either a digital or balance-beam scale. Body mass index (BMI) was calculated as weight (kg)/height (m^2^). Age- and gender- independent height and weight z-scores were calculated as previously described, using the Ped(z) app with the WHO reference intervals (16).

Blood samples were collected in anticoagulant and lithium heparin tubes. Creatinine was measured using the calorimetric modified Jaffe method with alkaline picrate (17) and were isotope dilution-mass spectrometry traceable. Estimated GFR (eGFR) was calculated from the average of the three height measurements and one serum creatinine with the new modified Schwartz formula (18). Fractional excretion of sodium excretion was also calculated in percent using 100*(urinary sodium * serum creatinine)/(urinary creatinine * serum sodium) (19).

Urinary KIM-1 was measured using Luminex® technology immunoassay. Antibodies were obtained from R and D systems. The assay was run according to standardized protocol. For KIM-1 the lower and upper limits of quantitation on a seven-point calibration curve were 1.22 and 5000 pg/ml, respectively. Intra- and inter-assay precision were <6%, recovery of spiked samples was 104%–107%, and dilutional linearity was shown at 1 in 10, 1 in 100 and 1 in 1,000 dilutions in the assay diluent.

### Statistical analysis

Anthropometric z-scores were calculated using the app Ped(z) based on WHO growth charts (16). Renal volume z-scores were calculated using the same app based on the body surface area nomograms based on Scholbach and Weitzel (20).

Calculations were always performed based on the mean of all repeated measurements. Continuous variables were analyzed for normal distribution using the D'Agostino-Pearson normality test (21). We used descriptive statistics wherever possible. For continuous variables we used linear regression throughout as most of the parameters were normally distributed. We also used multivariate regression with urinary calcium, serum sodium, renal length and gestational age as independent variables and urinary KIM-1 as dependent variable. No adjustments were made for missing data. All calculations were performed using GraphPad Prism version 5.0 for Mac, registered to GF, or STATA 14.0 for PC, registered to Fundación Valle de Lili.

## Results

### Patient recruitment

**Figure 1:**
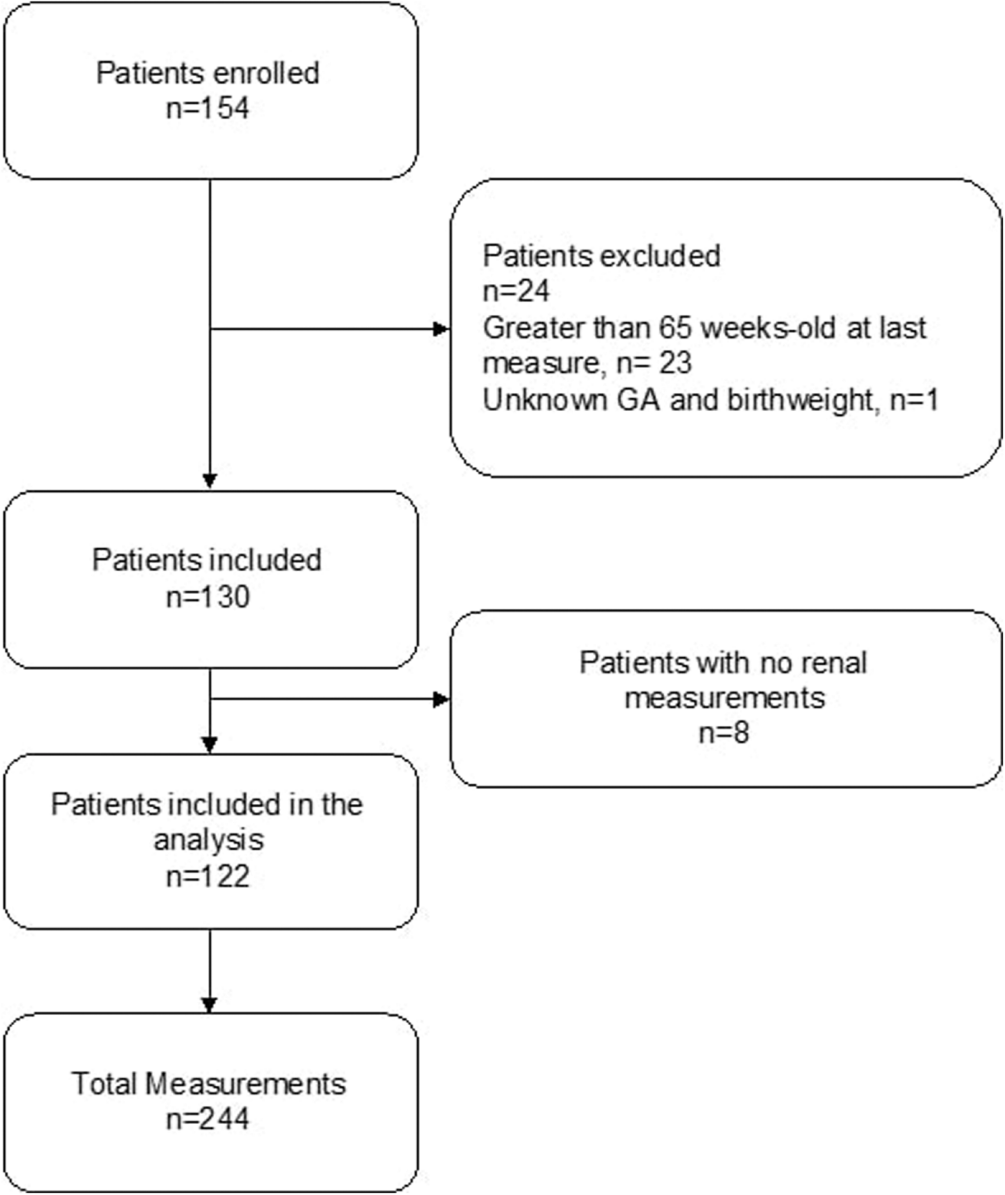
Flowchart of the patients included.

Figure 1 demonstrates the flow of the patients included. Of the 154 eligible patients approached, 129 could be included into the study (32 full-term controls and 97 pre-term participants). Table 1 summarizes the patient characteristics.

**Table 1:** Demographics, renal volume and renal function measurements according to gestational age.

Characteristics	Term five-years-old children *n* = 33	Preterm five-years-old children *n* = 97	*p* value
Gestational age	38 (38–40)	32 (30–33)	<0.001
Preterm classification, *n* (%)			
Less than 28 WGA	−	17 (17.5)	−
29–32 WGA	−	49 (50.5)	−
33–36 WGA	−	31 (32)	−
Age [years]	4.9 (4.8–5.1)	5.0 (4.9–5.1)	0.224
Birthweight [grams]	3,250 (3000–3600)	1,470 (1100–1795)	<0.001
Birthweight z-score	−0.35 (−1.27; 0.31)	−0.65 (−1.17; −0.19)	0.193
Average R renal length [mm]	73 (69.3–77.2)	70 (67.7–77.0)	0.357
Average L renal length [mm]	72.7 (68.7–76)	70.1 (67–73.7)	0.071
Average R renal volume [ml]	43.8 (35.8–58.5)	38.2 (33.5–44.6)	0.050
Average L renal volume [ml}	40.8 (37.7–49.9)	40.4 (35.1–47.8)	0.413
Average weight [kg]	18.47 (17.17–21.17)	17.1 (15.73–19.0)	0.005
Weight z-score	0.41 (−0.56; 0.41)	−0.24 (−1.05; 0.58)	0.041
Average height [cm]	107.8 (105.1–110.5)	105.9 (102.7–109.2)	0.096
Height z-score	0.05 (−0.59; 0.44)	−0.20 (−0.92; 0.37)	0.558
BMI [kg/m^2^]	16.0 (15.4–17.6)	15.4 (14.4–16.5)	0.008
BMI z-score	0.52 (−0.03; 1.41)	0.04 (−0.95; 0.78)	0.008
Weight/Height	18.5 (17.2–21.2)	17.1 (15.7–20.7)	0.004
Weight/Height z-score	0.44 (−0.07; 1.28)	0.11 (−0.94; 0.76)	0.020
Average R renal length z-score	0.93 (0.44; 1.32)	0.56 (0.13; 1.28)	0.121
Average R renal vol z-score	−0.59 (−1.08; −0.24)	−0.69 (−1.10; −0.23)	0.348
Average L renal length z-score	0.93 (0.37; 1.32)	0.82 (0.21–1.40)	0.588
Average L renal vol z-score	−0.71 (−1.02; −0.09)	−0.52 (−0.99; −0.02)	0.646
Serum creatinine [mg/dl]	0.35 (0.32–0.41)	0.34 (0.31–0.39)	0.415
Serum creatinine [µmol/l]	30.4 (28.2–35.8)	30.4 (27.4–34.4)	0.463
Schwartz eGFR [ml/min/1.73 m^2^]	104.9 (91.3–112.3)	102.8 (93.9–110.8)	0.932

### Preterm and control groups comparisons

The results of all measurements are summarized in Table 1 for the entire cohort. We compared parameters between five-years-old pre-term patients and healthy age-matched controls. While gestational age was significantly different between the pre-term babies (median 32 weeks, range 25–36) and the healthy full-term controls (median 38 weeks, range 37–42), *p* < 0.0001, Mann-Whitney test), we found significantly lower weight and body surface area between the pre-term and full-term children at age 5-years. Blood pressure measurements, blood pressure z-scores, serum creatinine, eGFR, renal volumes and renal volume z-scores did not differ significantly between pre-term and full-term participants at 5 years of age. The only exception was the right renal length, but not the right renal length z-score.

Median urinary KIM-1 was 17.8 pg/ml (interquartile range 4.6, 59.0 pg/ml) in the pre-term group and was not significantly different from the full-term group at 5-years (median 17.3 (interquartile range 12.3, 50.7 pg/ml).

There was a trend towards higher urinary protein in the pre-term group. Median urinary protein concentration was 7.35 mg/dl (IQR 4.57, 11.39) in the pre-term group and 5.54 mg/dl (4.01, 7.81) in the control group, *p* = 0.0745. There was a trend towards higher urinary creatinine in the pre-term group, median creatinine concentration was 49.9 mg/dl (IQR 24.4, 81.6) in the premature group and 34.2 (19.9, 57.85), *p* = 0.0879. However, urinary sodium did not differ. The urinary sodium/potassium ratio and the fractional sodium excretion did not differ between the groups. There was a trend towards higher urinary calcium in the pre-term group. In the pre-term group, median calcium concentration was 5.98 mg/dl (IQR 1.70, 10.4) and 3.16 (1.39, 6.65) in the control group (*p* = 0.0523). The urinary calcium/creatinine ratio, and urinary pH however, did not differ between the groups.

### Spearman correlation of urinary KIM-1 and patient measurements

Table 2 describes the spearman correlation between urinary KIM-1 and the other measurements taken from the patients. In patients full-term five-years old patients, average left renal length and serum chloride had a strong correlation with serum KIM-1 concentration (*n* = 7 Spearman 0.786, *p* = 0.036). In moderate preterm patients, there was a moderate correlation with z-score for height and KIM-1 (*n* = 33, Spearman 0.408, *p* = 0.020). In the 5-year extreme preterm patients, diastolic blood pressure z-score was strongly correlated with serum KIM-1 concentration (*n* = 11, Spearman 0.770. *p* = 0.004). There was no correlation between gestational age at born and KIM-1 serum concentration (*n* = 73, Spearman −0.081, *p* = 0.423).

**Table 2:** Spearman correlation of urinary KIM-1 with individual measurements according to gestational age.

Parameters	All patients	Term patients	Late preterm patients	Moderate preterm patients	Extreme preterm patients
	*n* = 73	*n* = 7	*n* = 22	*n* = 33	*n* = 11
Weeks of Gestational age	−0.0805	−0.339	0.043	−0.175	0.162
Average weight [kg]	0.1927	0.643	−0.156	0.256	−0.109
Weight z-score	0.1274	0.607	−0.174	0.316	−0.041
Average height [cm]	0.2956	0.179	−0.156	0.311	−0.324
Height z-score	0.152	0.286	−0.184	0.408*	−0.214
BMI [lg/m2]	0.3029	0.536	−0.167	0.166	−0.025
BMI z-score	0.1084	0.429	−0.124	0.093	−0.091
Weight for Height	0.1596	0.643	−0.116	0.258	−0.182
Weight for Height z-score	0.1229	0.536	−0.169	0.108	−0.041
Average R renal length [mm]	0.0921	0.523	−0.053	0.160	−0.159
Average L renal length [mm]	0.0078	0.786*	0.187	0.190	−0.218
Average R renal volume [ml]	0.1671	0.429	−0.226	0.178	−0.574
Average L renal volume [ml]	0.1627	0.429	−0.132	0.253	−0.092
Average R renal length z-score	0.0762	0.357	−0.007	0.122	−0.143
Average R renal vol z-score	0.1295	0.214	−0.213	0.093	−0.353
Average L renal length z-score	0.1295	0.536	0.189	0.118	−0.160
Average L renal vol z-score	0.1295	−0.036	0.015	0.246	−0.008
Hemoglobin [mg/dl]	0.2488	0.018	0.150	0.203	0.593
serum sodium[mmol/l]	−0.0709	−0.185	−0.376	0.243	−0.399
Serum potasium [mmol/l]	0.2156	0.714	−0.066	0.289	0.447
Serum Cl [mmol/l]	0.0991	0.786*	−0.228	0.176	0.009
Serum Mg [mmol/l]	−0.0945	−0.036	−0.259	0.175	−0.360
Serum Ca [mmol/l]	0.0534	0.429	−0.105	0.097	0.046
Serum BUN [mg/dl]	0.0472	−0.143	0.046	0.197	0.286
Serum *P* [mg/dl]	0.1444	0.107	−0.081	0.254	0.383
Serum Creatinine [umol/l]	0.0542	0.054	0.083	0.207	−0.193
Serum Creatinine [umol/l]	−0.0112	0.054	0.083	0.207	−0.193
Schwartz eGFR [ml/min/1.73 m^2^]	0.0734	−0.107	−0.142	−0.020	0.150
Urine protein [mg/dl]	−0.22	−0.071	−0.346	−0.223	−0.100
Urine Creatinine [mg/dl]	−0.136	0.000	−0.270	−0.116	0.205
Urine Na [mmol/l]	0.002	0.607	−0.256	0.091	0.100
Urine K [mmol/l]	−0.0454	0.000	−0.360	0.096	0.535
Urine Ca [mmol/l]	−0.0176	0.643	0.207	−0.194	−0.046
Urine Ca/Cr [mmol *dl/mg]	0.0761	0.536	0.422	−0.072	−0.114
Urine specific gravity [mg/ml]	−0.0087	0.090	−0.047	−0.039	0.085
Urine pH	−0.0849	−0.146	−0.129	−0.064	0.087
Systolic blood pressure [mmHg]	−0.1065	−0.429	0.010	0.019	0.466
Systolic blood pressure z-score	0.044	−0.143	−0.075	−0.078	0.451
Diastolic blood pressure [mmHg]	−0.067	0.090	0.112	−0.047	0.770*
Diastolic blood pressure	0.1765	0.321	−0.004	−0.001	0.770*

R, right; L, left; Na, sodium; K, *p*otassium; Cl, chlorine; Mg, magnesium; Ca, calcium; P, phosphorus; eGFR, estimated glomerular filtration rate.

*p-value < 0.05.

BMI, body mass index; L, left; R, right; vol, volume; BUN, urea nitrogen; Ca, calcium; Cr, creatinine; KIM-1, kidney injury molecule 1, significant parameters are marked by a darker shaded square box around the parameter.

## Discussion

In this ambispective study of pre-term born 5-year-old children and healthy full-term controls, we found that KIM-1 was not related with gestational age and birth weight. Also, urinary KIM-1 concentrations did not differ between pre-term and full-term born at age 5-year-old. We found no differences between both groups for the large array of other clinical, ultrasound and biochemical tests assessing renal function, including fractional sodium excretion. No other biochemical or clinical or radiological parameter gave a signal that could be related to lower nephron endowment. We must reject the hypothesis that urinary sodium excretion was related to gestational age in 5-year-old children born prematurity.

Developmental programming of a reduced nephron endowment is more than just a infant's birth weight (22). About 30 years ago, David Barker postulated that low birth weight is associated with increased cardiovascular morbidity and mortality in adults, which was later linked to renal disease (23). The hypothesis of developmental origin and fetal programming of adult disease is considered proven and low nephron endowment may play a major role in this association (24). As outlined in the introduction, nephrogenesis ceases at 36 weeks of gestation and is likely reduced with premature delivery (2). To date, we don't have a precise tool for estimating nephron endowment *in vivo*. Experimental methods using magnetic resonance imaging in mice (25) or in rabbits (26) have not yet been established in humans. Effective renal plasma flow with concurrent measurement of glomerular filtration rate may reveal hyperfiltration and thus reduced nephron endowment (27); however, nuclear medicine measurements of effective renal plasma flow remain available only in the research setting. A biomarker has been lacking. KIM-1 seemed to be positioned as a promising biomarker to detect chronic kidney failure promptly (28). However, in this paper, there was no correlation between urinary KIM-1 concentrations and gestational age in five-year-old children; therefore KIM-1 could not be a surrogate for renal tubular dysfunction at five years of age. Our findings agree with a similar sized study from India where there was also no difference between 100 low birth weight and 66 normal birth weight children at three different time points during the dynamic phase of renal maturation in infancy (29). Similarly Askenazi et al., in a prospective cohort study on 113 VLBW infants (weight < 1200 g or <31 weeks' gestation) using urinary biomarkers, didn't find KIM-1 as an early tubular marker in patients with AKI neither without AKI (30).

Our study limitations include the sample size. Only 73 patients had urinary KIM-1 measured, of which only 7 patients were born at term. Future, larger studies must confirm these empirical findings. There were challenges with the recruitment of the control group and the healthy children do not have prospective data in this ambispective study. The data are also limited to predominantly Latino children and while a small proportion of patients were African descendants (11.5%, data not shown), the number was quite small to perform a subgroup analysis. Moreover, there was significant attrition in the prospectively recruited premature cohort, which introduces an unclear bias. The data may therefore not be generalizable and certainly must be confirmed in other ethnic backgrounds. Also, other urinary tubular markers such as Neutrophil gelatinase-associated lipocalin (31) and biomarkers of glomerular filtration rate such as cystatin C (32) were not employed.

Iyengar's study also did not demonstrate any differences when measuring estimated GFR with cystatin C (29). Strengths of the study include the wide array of clinical, anthropological, biochemical, and imaging modalities to assess these patients. The prospective nature of the prematurely born cohort is another strength.

Taken together, we found that urinary KIM-1 was not significantly correlated with gestational age and birth weight in a cohort of 5-year-old children born at term and prematurely, our findings need to be confirmed in a larger prospective study.

As is known, renal growth in both cortical and medullary parts increase significantly during pre and adolescence. Our study aimed to evaluate the impact of prematurity on renal function measured with urinary KIM-1 at the middle age of 5 years. Although this study did not show significant differences, it is a starting point for adolescent prospective evaluation, age of high metabolic demand. The metabolic challenge generated by body growth and the requirement for kidney growth added to the history of prematurity can be reflected in the tubular injury expressed in alterations in markers such as KIM-1, NGAL and cystatin C.

## Data Availability

The data that support the findings of this study are available on request from the corresponding author, LTC. The data are not publicly available due to the international ethical regulations that protect the data security of the subjects.
